# Puberty and Menstruation Knowledge, Information Sources and Needs among Secondary School Adolescent Girls and Boys in Kibaha, Tanzania

**DOI:** 10.1371/journal.pgph.0004176

**Published:** 2025-03-10

**Authors:** Judith Msovela, Angela E. Shija, Hyasintha Ntuyeko, Calister Imeda, Aidat Mugula, Erick Mgina, Annastazia A. Egidio

**Affiliations:** 1 National Institute for Medical Research, Dar es Salaam, Tanzania,; 2 KASOLE Secrets Co. LTD, Dar es Salaam, Tanzania,; 3 Tanzania Horticultural Association, Zanzibar, Tanzania; Tata Institute of Social Sciences, INDIA

## Abstract

This baseline study aimed to assess the knowledge, information sources, and needs of adolescent boys and girls regarding puberty and menstruation. The study was conducted in selected schools before establishing school health clubs. The objective was to gather information that would inform the development of puberty education programs tailored to the specific needs of the adolescent population. A cross-sectional study was conducted in Kibaha Town council from 17th to 31st March, 2020 involving both adolescent boys and girls. Data was collected using a combination of quantitative and qualitative methods, including a semi-structured questionnaire and focus group discussions (FGDs). Quantitative data was analysed using descriptive statistics while qualitative data was analysed using a thematic approach. The study involved 591 students, with 461 completing a questionnaire and 130 participating in FGDs. Results showed that many adolescent boys and girls had limited knowledge about puberty and menstruation. Only about 34% of participants felt well informed about puberty, and 31% about menstruation. Participants’ knowledge of specific aspects ranged from 36.2% to 97.4% for puberty and 21.7% to 87.4% for menstruation. Participants had inadequate knowledge of issues such as age at puberty, some physical changes in boys, the mechanism of menstruation, and the menstrual cycle. Before menarche, 39% of respondents primarily relied on schoolteachers for information about menstruation. However, this shifted significantly after menarche, with 51% of respondents citing their mothers as the primary source. However, students preferred to get information from school clubs (40%), health care providers (23%) and school teachers (11%). Further, very few students indicated receiving information before puberty. Girls indicated the need for more information as compared to adolescent boys, particularly on issues related to menstrual hygiene, the use of menstrual materials, and how to manage menstrual pains. This study reveals significant gaps in adolescents’ knowledge of puberty and menstruation, underscoring the need for comprehensive and early education. School health clubs, particularly when facilitated by external health professionals and integrated with WASH programs, offer an effective platform for addressing these gaps, providing a comfortable space for learning and empowering both boys and girls with critical knowledge and skills. Empowering both boys and girls through such initiatives can reduce stigma, foster supportive behaviors, and promote adolescent well-being. Findings were used to inform the development of materials for the facilitation of school clubs, for both girls and boys.

## Background

Puberty is the process of physical maturation, where an adolescent reaches sexual maturity and becomes capable of reproduction. It is a transitional period between childhood and adulthood, characterized by changes in the body, brain, behaviour, cognition, and emotion [1]. Puberty education is essential during the early adolescence stage to help both boys and girls manage their physical, emotional, and interpersonal changes during the transition to adulthood [1,2]. The puberty education also helps them cope and handle challenges with intensifying peer and societal pressures that arise during puberty positively, in healthy ways and with less fears [3]. Accurate information and support for young people can empower them to make informed choices about their health and well-being. Girls’ knowledge before their first menstruation helps them to reduce fear, embarrassment, and shame and manage their menses well while in and out of school [4,5].

Education about puberty is a crucial aspect of adolescent development [6]. Young adolescent girls in low-middle-income countries are under-prepared for puberty and menstruation [7]. Studies have shown that many adolescents in the region such as in Nigeria, Kenya and Uganda lack adequate knowledge about puberty, leading to misconceptions, anxiety, and stigmatization [1,8,9]. This lack of knowledge can contribute to feelings of confusion, shame, and isolation [10] which may lead to mental, and emotional stress and challenges if not addressed timely [11]. Inadequate knowledge about menstruation among girls can negatively impact their social interactions during their menstrual periods [7]. Studies conducted in developing countries such as in Pakistan and Indonesia indicate only a few adolescents have adequate knowledge of menstruation [12,13]. Managing menstruation in hygienically with dignity is a matter of health and human right, hence girls need comprehensive and correct information before and after menarche. Inadequate knowledge, skills and support during menstruation can affect girls’ participation in terms of attending school and participating in school activities.

UNESCO recognizes that education about puberty is a cornerstone of adolescent development [6]. However, young adolescent girls in low- and middle-income countries (LMICs) are often ill-prepared for the onset of puberty and menstruation. This lack of knowledge has significant consequences, including an inadequate understanding of future fertility implications, which can negatively impact their psycho-social well-being [14].

Studies across various LMICs reveal a concerning lack of pre-menarcheal knowledge. In Bangladesh, Bosch and colleagues reported that 64% of girls experienced menarche with fear. Similarly, a study in India, found that 60.3% of girls were unaware of menstruation before its onset, demonstrating a poor understanding of its physiological processes [15]

While mothers often serve as the primary source of information, studies indicate that this is not always the preferred method for girls. A study in Nepal, found that 65.3% of girls preferred learning about puberty from textbooks [16]. Turkish research revealed that over half of schoolgirls preferred receiving puberty education from health professionals, while a third favored information from their families. Only 5.9% of girls preferred teachers as their primary source of puberty education [17].

These observations highlight the social stigma surrounding menstruation, with adolescents often viewing discussions about menarche as socially unacceptable and rude [18]. This stigma is further evident in cultural restrictions surrounding menstruation. A study in Kenya reported that menstruation is often viewed as a deeply private and secretive bodily function [19]. Similarly, a study in Nepal, found that 70.7% of girls believed they could not attend school during menstruation, and 100% believed they were restricted from cooking [16]. In Tanzania, while puberty education is integrated into the primary and secondary school curriculum [20,21], crucial information on menstrual health, hygiene and management is largely absent.

This gap in education leaves most adolescents ill equipped to navigate the physical and emotional changes associated with puberty. The National Guideline for Water, Sanitation, and Hygiene for Tanzania Schools acknowledges this critical omission [22].

School clubs offer valuable platform for adolescents to learn about the physiological and emotional changes during puberty and receive practical guidance on menstrual health management. These clubs can enhance knowledge about puberty and menstrual health among adolescents, particularly in settings where these topics are stigmatized or poorly understood. Furthermore, engaging boys in these clubs promotes inclusivity and reduces gender-based stigma surrounding menstruation.

Source of information on puberty and menstruation is also a concern to adolescents. A previous study in Tanzania demonstrated that adolescents may find puberty and menstrual health education more acceptable when delivered by external facilitators such as trained health professionals or community health workers [23]. Studies conducted in Tanzania and Uganda, show that adolescents feel more comfortable discussing menstrual sexual and reproductive health issues with adults who are not their teachers [23,24].

This highlights the importance of collaborative efforts with health professionals to provide practical skills, such as tracking menstrual cycles and pain management and empower young people to make informed decisions about their health.

Furthermore, research from Nepal, Iran and India has demonstrated that participation in school-based programs significantly improves adolescents’ knowledge, attitudes, behaviors, and practices related to menstrual hygiene [25–27].

The school club platform provides an ideal setting for expert facilitation, interactive learning experiences (such as discussions and hands-on activities), and access to accurate information. The implementation of school clubs aligns with the global ten priorities (2014–2024) for creating evidence for scaling Menstrual Hygiene Management (MHM) in schools, as outlined by Sommer [28]. This framework emphasizes the importance of innovative approaches like school clubs to improve menstrual health among adolescents.

There is no adequate information in the country on the level of adolescents’ knowledge of puberty and menstruation and information needs. A study conducted in Northern Tanzania showed a significant gap in puberty information, particularly among girls (16). There are disparities in access to various information platforms between rural-urban, the poor and the rich, which is also shaped by social, cultural values, beliefs and family background [29]. Hence, it is important to understand the best options to deliver puberty and menstrual information to school adolescents. Therefore, this study was conducted to understand the status of knowledge of puberty and menstruation, information sources and needs in both adolescent boys and girls, to inform the implementation of the school clubs in project schools.

## Methods

### Study design and settings

We conducted a cross-sectional study employing mixed methods in data collection from 17th to 31st March 2020. Data collection involved quantitative and qualitative methods using a guided self-administered semi-structured questionnaire and focus group discussion (FGD) guide.

This report is part of a baseline study for a Public Private Partnership project on menstrual health that was conducted in Kibaha Town council of Pwani region. The study was conducted in six public secondary schools which include Simbani, Mwambisi Forestry, Bundikani, Pangani, Visiga and Kibaha Girls. All were mixed schools except Kibaha was for girls only. Public schools were selected for the project implementation because the 2018 school water, sanitation and hygiene (WASH) assessment report shows they are disadvantaged more than twice in terms of WASH facilities and infrastructure which affects menstrual management in schools [30].

### Study population, sampling and sample size determination

The study involved secondary school adolescent boys and girls from Form one (class/grade 8) to Form three (class/grade 10) who were selected using simple random sampling technique on the survey day.

The sample size estimation for survey participants was obtained by a formula for a single proportion in cross-sectional studies, with a proportion of 50%, at a margin of error of 5% (d = 0.05) and confidence level of 95%(a = 0.05), the obtained sample size was 384 and added 10% for the non-response to a final sample size of 422. The selection of survey participants gave more chances for girls than boys, with a ratio of 3:1, since most of the established school clubs in the past projects were attended by girls only due to the nature of the projects which focused on menstrual health and hygiene in schools.

On the other hand, participants for focus group discussions (FGDs) were purposively selected. In each school, two FGDs where conducted, one for boys and the other for girls. We conducted a total of 11 FGDs, each group comprised of 12 participants.

### Data collection

Mixed methods were used, which allowed a comprehensive understanding of the research topic by capturing both quantitative and qualitative aspects. For quantitative data, a semi-structured and guided self-administered questionnaire was used to collect data on demographic characteristics, knowledge of puberty and menstruation and needs. For qualitative data collection, an FGD guide was used to obtain participants’ knowledge, experiences, attitudes and recommendation.

### Data management and analysis

Quantitative data was entered in Epi-Data software version 4.1 and analysis was conducted using Stata version 14.0 (STATA Corp, Texas-USA). Descriptive analysis was conducted to assess prevalence and distributions, while the Pearson Chi-square statistical test was used to compare group differences for categorical variables. Relationship, association, and difference between variables are considered statistically significant if P < 0.05. Qualitative data was managed manually, and thematic analysis was applied based on predetermined themes.

### Ethical considerations

Ethical clearance was obtained from the National Health Ethics Review Committee (NatHREC) (approval number NIMR/HQ/R.8a/Vol.1X/3368). Permission to conduct field data collection was granted by the local government authorities and the school management. Written consents were signed by the school health teacher of each respective school on behalf of parents for all students participating in the study since the study was linked to the school health program. Additionally, all students who agreed to participate in the study signed a written assent form before conducting the survey after adequate information about the study was given. All participants were allowed to participate in the study voluntarily and were free to withdraw at any time without being penalized.

## Results

### Demographic characteristics of participants

A total of 461 secondary school students completed a self-administered questionnaire. Female students constituted nearly two-thirds (70.3%, n = 324) of participants. The mean age of all respondents was 14.7 ± 9 years and didn’t differ between girls and boys. Respondents from each class constitute a third of the overall participants (Table 1). Additionally, a total of 130 students, 55.4% (72) girls and 44.6% (58) boys from the six project schools participated in FGDs. A total of 11 FGDs were conducted, 6 for girls and 5 for boys.

**Table 1:** Demographic Characteristics of Survey Participants.

Variable	Number (%)
**Sex**	
Girl	324 (70.3)
Boy	137 (29.7)
**Age categories**	
12–14	218 (47.3)
15–19	243 (52.8)
**Class Level**	
Form I	150 (32.5)
Form II	143 (31.0)
Form III	168 (36.4)

### Self-assessment on knowledge of puberty and menstruation among students

Students were asked if they have adequate knowledge of puberty and menstruation. To their level of self-assessment of knowledge on puberty, about one-third (34.1%) of students acknowledged having adequate knowledge on puberty with a higher proportion of boys (43.8%) reporting to have adequate knowledge compared to girls (29.9%). There was a higher proportion of older students (aged 15–19 years) who perceived themselves to have an adequate level of knowledge compared to the younger students (12–14 years). Additionally, there was a difference in adequate knowledge among students across the schools (Table 2). Regarding the level of knowledge on menstruation, similarly around a third (31.2%) of students felt that they have adequate knowledge of menstruation, with girls (36.1%) having higher proportion of those perceiving to have adequate knowledge compared to boys (19.7%).

**Table 2:** Self-assessment of knowledge on puberty and menstruation.

	Girls				Boys			
Characteristics	Knowledge on puberty		Knowledge on menstruation		Knowledge on puberty		Knowledge on menstruation	
	Yes (%)	No (%)	Yes (%)	No (%)	Yes (%)	No (%)	Yes (%)	No (%)
**Overall**	**97 (29.9)**	**227 (70.1)**	**117 (36.1)**	**207 (63.9)**	**60 (43.8)**	**77 (56.2)**	**27 (19.7)**	**110 (80.3)**
**Age categories**								
12–14	41 (25.8)	118 (74.2)	49 (30.8)	110 (69.2)	23 (39.0)	36 (61.0)	11 (18.6)	48 (81.4)
15–19	56 (33.9)	109 (66.1)	68 (41.2)	97 (58.8)	37 (47.4)	41 (52.6)	16 (20.5)	62 (79.5)
**School**								
Bundikani	12 (21.4)	44 (78.6)	17 (30.4)	39 (69.6)	13 (54.2)	11 (45.8)	9 (37.5)	15 (62.5)
Mwambisi forestry	15 (37.5)	25 (62.5)	14 (35.0)	26 (65.0)	15 (41.7)	21 (58.3)	3 (8.3)	33 (91.7)
Pangani	22 (41.5)	31 (58.5)	24 (45.3)	29 (54.7)	13 (56.5)	10 (43.5)	3 (13.0)	20 (87.0)
Simbani	9 (17.3)	43 (82.7)	18 (34.6)	34 (65.4)	13 (46.4)	15 (53.6)	8 (28.6)	20 (71.4)
Visiga	10 (18.5)	44 (81.5)	13 (24.1)	41 (75.9)	6 (23.1)	20 (76.9)	4 (15.4)	22 (84.6)
Kibaha girls	29 (42.0)	40 (58.0)	31 (44.9)	38 (55.1)	NA	NA	NA	NA

### Knowledge of puberty

During the survey, students were asked about specific puberty issues to assess their knowledge. The proportion of students who responded correctly to the statements related to puberty ranged from 36.2% to 97.4%. Specifically, at least 90% of students responded correctly to the following statements, puberty is the stage where a child’s body develops into adulthood capable of sexual reproduction (97.4%); increased body fat may play a role in regulating the onset of puberty; puberty is accompanied by emotional changes such as mood changes and sexual desire (92.6%); girls experience puberty early than boys (91.5%) and during puberty, hips broaden for girls (94.6%). At least 60% of participants responded correctly in the statement such as sign of puberty for girls is breast development (86.8%); during puberty, a girl’s body produces hormones called estrogen (71.4%); it is common for a female to have one breast positioned slightly higher than the other (63.1%); during puberty boys start to produce sperms and experience wet dreams (83.9%); testosterone hormone is responsible for physical changes in boys (63.1%). The statements that scored the lowest include boys’ breasts may enlarge during puberty (36.2%) and it is normal for one testicle to hang below the other in boys (42.5%).

During FGDs, students showed some general knowledge on specific issues of puberty and the level of knowledge varied across schools. In responding to a question about what puberty is, the majority of participants indicated that puberty is the transition from childhood to adulthood and it is associated with physiological, biological, and physical changes and the individual is capable of reproduction.

*“Puberty is a stage in which an individual moves from one stage to another, but it is associated with body changes”* (Girls FGD-5).

*“Puberty is the period where the child begins to mature physiologically, physically, biologically, socially and their bodies are capable for reproduction” (*Boys FGD-1).

*“From what I understand, puberty is a condition in which a boy or a girl reaches adulthood. There are several challenges they face such as having the desire to try things which are done by community members to see the outcomes, in doing so they suffer consequences such as early pregnancy and sexually transmitted infections”* (Boys FGD-5).

Some participants showed that they have an idea of hormonal changes during puberty. However, they had a wrong explanation of hormonal changes and pregnancy. One of the FGD participants had this to say:

*“For changes occurring in girls……… the girl begins to produce a certain amount of hormones, the levels become high leading to reproductive organs to produce hormones to receive male gametes which leads to pregnancy”* (Boys FGD-2).

Regarding the age in which puberty occurs, the common responses were at around 9–11 years in girls and 14 years for boys. However, there were also different responses concerning the age of puberty, with some mentioning the older ages as the common age when puberty occurs.

*“What I know is puberty occurs at 16 to 17 years, and if it doesn’t occur, then there might be a problem”* (Girls FGD-5).

*“Puberty for females begins at 11 to 14 years, while for males, it happens from 14 to 18” (*Boys FGD-1).

The participants could also mention characteristics of puberty for both boys and girls. For girls, changes like the development of breasts, pubic and armpit hair, soft voice, soft and glowing skin, broadening of hips and menstruation were mentioned by some discussants. For boys, the development of beard, pubic and armpit hair, deep voice, broadening of the chest, mood changes, and having wet dreams. Further, in some focus groups, attraction to the opposite sex and desire for sex were also mentioned.

*“During puberty some boys and girls like to have sexual intercourse” (*Girls FGD-1).

*“Due to emotional changes associated with puberty, both boys and girls are attracted to the opposite sex, and they develop a desire for sexual intercourse especially when they see the opposite sex…. a boy will be attracted when he sees a girl’s breast and broadened hips”* (Boys FGD-1).

From the FGDs it appears that more emphasis of puberty information is put on girls while boys receive little or no information.

*“Boys have not been prepared…. more emphasis of puberty education is put on girls”* (Boys FGD-3).

### Knowledge of menstruation

The survey results show students had different levels of knowledge of various issues of menstruation, with 21.7% to 87.4% of participants providing correct responses to aspects of menstruation. The majority of students (82.7%) could identify that menarche starts between 10 and 15 years; menstrual cycles range from 21 to 35 days-(43.4%); the menstrual cycle are counted from the 1st day of the period to 1st day of next period (57.1%); menstrual flow takes an average of 3–7 days (78.3%); it is normal for young girls to skip periods for 30–60 days (27.1%); It is recommended to change menstrual pads at least every 4 to 8 hours (74.2%); improper use of menstrual materials may contribute to reproductive tract infections, e.g., bacteria or fungus infection (82.9%); girls or women should change the menstrual materials at least 3 times a day (87.4%); light exercises can help reduce pain during menstruation (73.3%) and bathing with warm water or using hot pads helps to relieve menstrual cramps (58.6%).

Findings of FGDs, similarly reflected a difference in adolescents’ knowledge of menstruation; some participants appeared to be more knowledgeable of different aspects while others had partial knowledge about menstruation. For example, some of participants were able to explain what menstruation is, but provided incomplete information about how the bleeding occurs. One pointed out that bleeding occurs after the ovum is released but failed to explain other issues that happen, leading to menstruation. Others associated the menstruation period with an increased desire for sexual intercourse.

*“When a girl is in menstruation becomes in danger days and is at risk of desire for sexual intercourse”* (Boys FDG-4).

Further, regarding the duration of menstrual bleeding, there were different answers, the common answers were 3, 4, 5, 8, and 9 days, with 9 days being mentioned by more participants. Responses on the duration of the menstrual cycle also varied with 28, 30, 31 and 35 being common responses. There was also confusion about the frequency of menstruation. From one FGD discussion, participants mentioned that menstruation occurs twice per month, in between the months and again at the end of the month.

*“From what I understand menstruation is when a girl is bleeding after puberty at the end of the month or in between the month”* (Girls FGD-1).

*“What I understand about menstruation is girls changing from a child to adulthood therefore menstruation may happen in between the month or at the end of the month”.* (Boys FGD-2).

Regarding symptoms which may occur during menstruation, participants had different opinions. Some of the participants, especially boys, describe menstruation to be associated with high fever, abdominal pain, and headaches. They went further to associate menstruation period with girls missing classes.

*“Things that happen when a girl is in menstruation include high fever, abdominal pain such that some of the girls do not attend school during menstruation”* (Boys FGD-2).

FGD findings further show that, there are still some myths and misconceptions related to the use of menstrual materials during period. An example of some misconceptions is the use of disposable sanitary pads. One of the FGD discussants narrates:

*“Some parents give their daughters pieces of cloth to use during menstruation and cautioning the girl that disposable pads are associated with side effects”* (Girls FGD1).

### Current and preferred sources of information on puberty and menstruation

**Fig 1:**
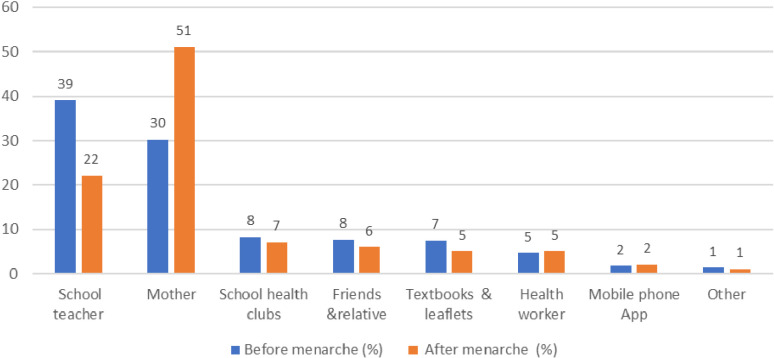
Sources of information on puberty before and after menarche.

Sources of information on puberty and menstruation varied before and after menarche. Teachers were mentioned by 39% as the main source of information before menarche, while mothers were mentioned by 51% as the main source of information after menarche (Fig 1).

During FGDs, participants highlighted that they receive puberty and menstruation information from teachers at school. The respective information is incorporated in school curriculum. One FGD participant reported,

*“Most of the time the information is obtained from school during the sessions. Teachers are not shy, they provide the information openly and, in some schools, they have schoolgirls programs where they provide the information”. Also, some students receive information from their parents before attaining menarche. The information provided is useful to prepare them for menarche” (*Girls FGD-4).

An additional source of information about puberty was from older siblings and relatives.

*“I first heard about puberty from my sister and brothers, they were talking about it as they were taught before, then when I reached class six……. I understood well about puberty, we were taught puberty topics while doing a science lesson”* (Boys FGD-2).

*“I was taught about puberty by my brother, my brother had a book about puberty, then when I reached class six, we were taught by our science teacher” (*Boys FGD-5).

Some of the FGD participants indicated the existence of school programs in some schools that provided health education including puberty and menstrual hygiene. They also acknowledged that the inclusion of puberty information in school syllabus is significant, especially considering the rapid advancements in technology.

*“For example, for us who come from Kongowe primary school we had a program of Room to Read, where we were taught about puberty and menstruation…. that is why we are knowledgeable….it was a program for form one only”* (Boys FGD-2).

**Fig 2:**
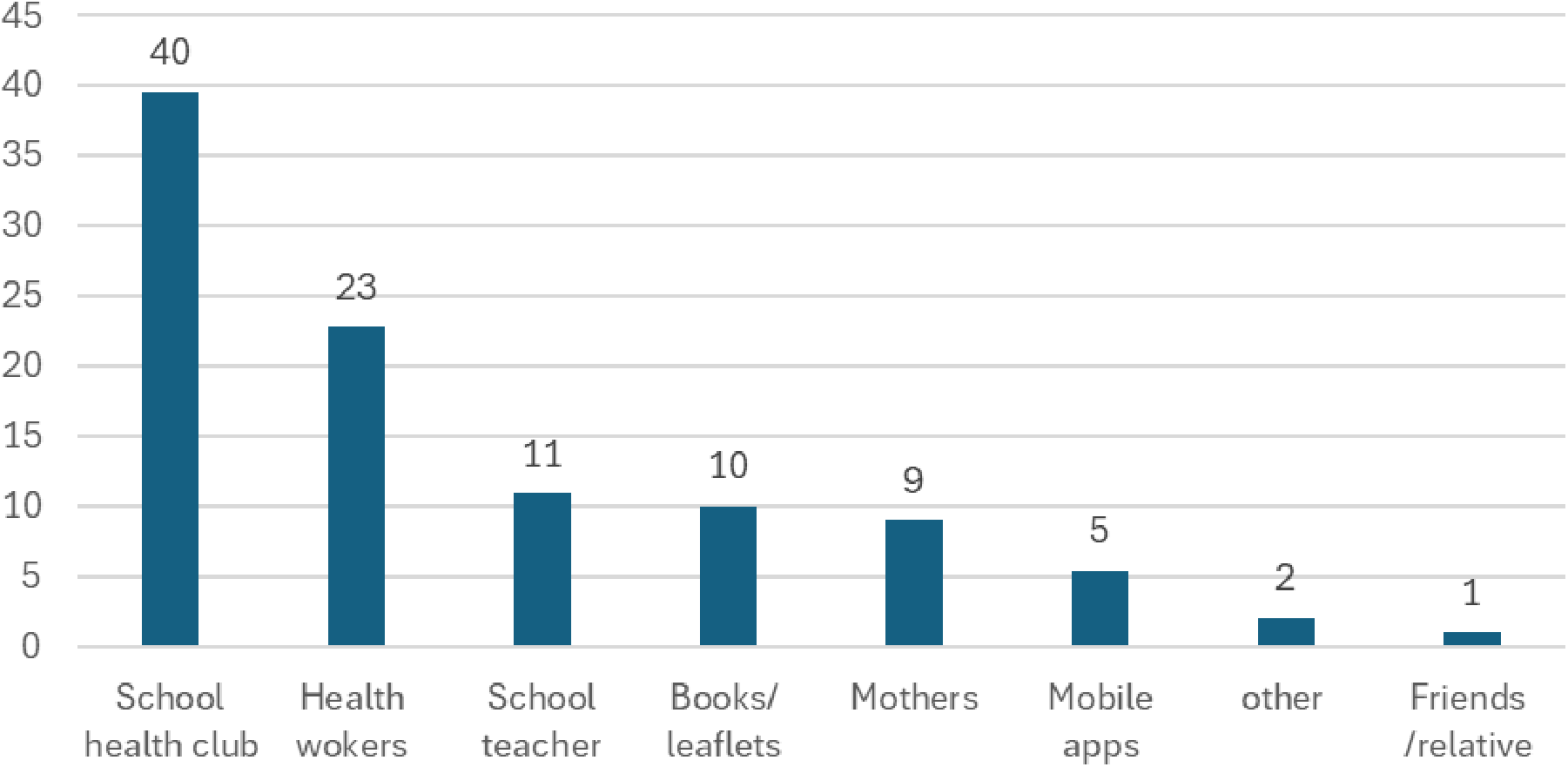
Preferred source of information on puberty and menstruation.

Survey respondents indicated a preference for school clubs (40%) as a source of puberty information, followed by health workers (23%), schoolteachers (11%), books and leaflets (10%) mothers (9%) and mobile apps (5%) (Fig 2).

During FGDs the preference for the source of puberty information mentioned differed from the survey results. Most FGD participants indicated that the preferred source would be from school teachers. The reason for their preference is a belief that teachers are more knowledgeable about biology in general. Also, they revealed that at school, sometimes there are seminars that provide this information to teachers. During FGD one student said:

*“The appropriate place to receive puberty information is at schools, because teachers are the ones knowledgeable of biology issues and also at school, they teach these topics in deep for the students to understand”* (Boys FGD-2).

Others suggested health facilities because parents are not capable of discussing issues considered taboos with their children.

### Timing of puberty and menstruation information

In FGDs it was revealed that most of the participants received information after attaining puberty. Often adolescents start searching for information after experiencing changes associated with puberty. They cited an example of a girl who, before menarche, may not be concerned by menstruation issues, but after starting menstruation, that’s where she starts to look for answers of what is happening to her and what to do. Additionally, at schools, puberty education is provided in classes five and six, the classes in which most adolescents have attained puberty. Most of the participants reported they received puberty information for the first time when they were in class five or six and at that time, they were aged 12 to 14 years.

*“A high percentage of adolescents receive information after puberty, because if an adolescent is in class six in which puberty education is provided but started class one at the age of 9 years then this adolescent would have been already passed the puberty stage by the time he/she reaches class six”* (Boys FGD-1).

Further, in home settings, most adolescents receive puberty information from their elders after the beginning of visible signs of puberty.

*“In our community, you may be sitting with your elders, they may tell you that they see changes happening to you as their child…they notice changes from one stage to the other, and from there they start to explain to you about puberty and caution you of things that you should avoid”* (Boys FGD-4).

Most of the FGD participants had the opinion that the best timing for puberty education is before puberty. They argued that providing the information before puberty will help the adolescent to prepare for the issues associated with puberty and know what to do with issues that arise. For example, in case of girls, when menarche occurs they can be able to take care of themselves, such as be able to prepare, use, and dispose of menstrual materials accordingly.

*“It would be better for adolescents to receive early puberty education. Providing prior information will help adolescents to be aware of issues to consider during puberty and will enable them to navigate the transition period smoothly”* (Boys FGD-1).

During FGDs, girls shared that, mothers and female guardians imparted puberty information after puberty, rarely before. Usually, girls are provided menstruation information after puberty. Among the reasons given for the late information is the discomfort of mothers to provide the information to their girls in adolescent phase due to fear that early information may lead to girls to conceal when they attain their menarche. One of the FGD discussants had this to say:

*“It depends on the comfort of the mother to provide information about puberty and menstruation…..for example, even grown-up girls who are in class six or seven do not receive information because the mothers feel that if they give them information prior to menarche, then when the girls reach menarche will not inform their mothers…therefore information is provided after menarche”* (Girls FGD-3).

While all focus groups indicated that some adolescents receive information about puberty before menarche, the extent of this information varied. For instance, only one or two participants reported receiving puberty information from their parents before their first menstruation. Similarly, a few participants mentioned receiving the information in class five, a time when most of them had not yet reached menarche. However, participants reported that some parents provide girls information about menarche, including what to expect during menstruation, appropriate menstrual materials and how to care for them.

*“Sometimes parent informs their grown-up girls before attaining their menarche on how to use pieces of cloths, and how to wash them after using, drying in the sun and after they dry to iron them, or they also instruct on how to use disposable sanitary pads and to ensure they stay hygienic throughout”* (Girls FGD-1).

### Puberty and menstruation information needs

Most participants in survey (93.5%) indicated that they need more information on puberty. During FGDs, both boys and girls indicated they needed more information about puberty and menstruation. Girls were interested to know about the menstrual cycle, why menstrual cramps occur, how to manage pain during menstruation, how to manage themselves during menstruation period and hygiene during menstruation. Also participants thought they needed more information on prevention of reproductive tract infections, prevention of early pregnancy and other issues during puberty.

In all FGDs, the participants highlighted that they need more education on puberty, and particularly, girls had some questions regarding menstruation, symptoms and hygiene, reasons for irregular menses and what to do about it and heavy menstrual flow.

## Discussion

This study was conducted to understand the status of puberty and menstruation knowledge to inform the implementation of the school program designed to improve understanding of puberty and menstruation.

About two-thirds of girls and boys have inadequate knowledge of puberty and menstruation. However, adolescent girls demonstrated a better knowledge of some aspects of puberty and menstruation compared to boys, particularly on physical and emotional changes occurring during puberty. Furthermore, most students have inadequate information about some aspects of puberty and menstruation. The study findings are in line with most studies in Africa, South Asia and other low and middle income countries that show low levels of knowledge [7,31].

Findings show girls had limited information on menstruation issues such as the menstrual cycle and hygiene management. This is in line with the scooping study conducted in middle and low-income countries, and Tanzania [32] which found limited information on menstruation issues among girls.

Findings indicate that the primary source of information about puberty is from teachers, followed by mothers. Some participants receive information about puberty from siblings and family members. This finding is similar to what was reported in other studies conducted in Tanzania where parents and teachers dominated as sources of information during menarche [32,33]. A similar trend was also shown in previous reports in other developing countries such as Uganda, South Africa and Pakistan [1,12,34]. Interestingly, over a third of participants indicated that they preferred receiving information on puberty from school health clubs where an external facilitator provides puberty education. This is in line with previous studies, which show that external facilitators are more acceptable to adolescents [23]. Adolescents feel more comfortable discussing menstrual, sexual, and reproductive health issues with adults who are not their teachers, especially in conservative contexts [23,24]. Moreover, findings show students prefer to receive information from health workers, because some of the issues they need to know more are not in school text books, hence, professionals can provide adequate information. Healthcare workers can use the school clubs to provide information to students; however, in all participating schools, none implemented a school health club, though the school timetable allocated one session per week for school club activities. School WASH guidelines [22] require all schools to establish WASH clubs that can be used as platforms for information sharing on puberty and hygiene practices.

Significantly few students indicated to receive information before puberty. As most students receive information from their teachers at school, this information is usually obtained after they have attained puberty, because puberty information is generally incorporated in science subjects in class five or six during primary education level. Similar findings have been observed in a study conducted in Kenya, which shows that although puberty and reproductive health topics are taught in upper primary, the timing is sometimes too late for those boys who have already started puberty [9]. These observations suggest a need for providing information about puberty earlier to ensure adolescents get the correct information before puberty.

Studies in Tanzania highlight the need for comprehensive interventions to improve menstrual health in schools like accessibility of menstrual materials and to improve awareness on mitigation menstrual related pain that leads to absenteeism and poor participation in classroom [23,35]. During FGDs, adolescent girls expressed a greater need for information compared to boys, particularly regarding menstrual hygiene, the use of menstrual materials, and managing menstrual pains. However, it is crucial to recognize that boys also benefit significantly from menstrual health education. With a better comprehension of menstruation, boys can become supportive figures to their female relatives and friends, challenging the stigma and taboos that often surround this natural bodily function [36]. Furthermore, as future parents, they will be better equipped to support their children during puberty and beyond [31].

Addressing the stigma and taboos surrounding menstruation is essential for creating a more inclusive and supportive environment for all individuals. Studies have shown that open discussions about menstruation can help to normalize the topic and reduce feelings of shame and embarrassment. For example, previous studies show that comprehensive sexuality education programs, which include information on menstruation, can help to challenge gender stereotypes and promote gender equality [37]. Equipping both girls and boys with accurate and age-appropriate information about menstruation, can create a more informed and supportive society where everyone feels comfortable discussing this important topic.

## Conclusion

This study highlights the significant knowledge gaps about puberty and menstruation among adolescents, emphasizing the need for comprehensive and early education on these topics. While adolescent girls demonstrated slightly better awareness of certain aspects of puberty and menstruation than boys, both groups showed significant knowledge deficits, particularly regarding menstrual hygiene and the management of menstrual cycles. The findings underscore the importance of providing accurate and timely information to both boys and girls to promote understanding, reduce stigma, and foster supportive behaviors.

School health club emerge as a promising platform to address these gaps effectively. An encouraging insight from this study is the expressed preference among adolescents for school health clubs as platforms for learning about puberty and menstruation. Such clubs, facilitated by external health professionals, offer a comfortable and reliable environment for students to discuss sensitive topics about puberty and menstruation and contribute to breaking taboos and promoting gender equity. Additionally, engaging boys in menstruation education is crucial, as it fosters understanding and supportive behavior, both in their current relationships and as future parents.

Establishing and utilizing existing school health clubs, particularly those integrated with school Water, sanitation and hygiene (WASH) program, can serve as effective platforms for providing timely, accurate, and comprehensive information on puberty and hygiene practices. These clubs could address the identified gaps, support early education before and after the onset of puberty, and empower both boys and girls with the knowledge needed to navigate puberty changes confidently.

Integrating school health clubs as a central component of puberty and menstruation education into existing education systems in collaboration with health workers, is a promising strategy for addressing these knowledge gaps, empower adolescents with important information and skills necessary to make informed decisions, manage challenges they face and promoting adolescent well-being.

## Supporting information

S1 Data Data set. (XLSX)
